# Supplementary material for: Natural soap is clinically effective and less toxic and more biodegradable in aquatic organisms and human skin cells than synthetic detergents
Source: PLoS One. 2025 Jun 18;20(6):e0324842. doi: 10.1371/journal.pone.0324842 (PMC12176228; doi:10.1371/journal.pone.0324842)
Supplement: Attachment — Submitted filename: renamed_652dc.docx [file pone.0324842.s003.docx]

Dear Editors and Reviewers

Thank you for reviewing our manuscript and offering valuable advice.

We have addressed your comments with point-by-point responses, and revised the manuscript accordingly.

**Responses to editor (Journal Requirements)**

***1. Please ensure that your manuscript meets PLOS ONE's style requirements, including those for file naming. The PLOS ONE style templates can be found at***

[***https://journals.plos.org/plosone/s/file?id=wjVg/PLOSOne_formatting_sample_main_body.pdf***](https://journals.plos.org/plosone/s/file?id=wjVg/PLOSOne_formatting_sample_main_body.pdf) ***and***

[***https://journals.plos.org/plosone/s/file?id=ba62/PLOSOne_formatting_sample_title_authors_affiliations.pdf***](https://journals.plos.org/plosone/s/file?id=ba62/PLOSOne_formatting_sample_title_authors_affiliations.pdf)

[Response]

We checked style templates and modified our manuscript and Figure file following it.

***2. To comply with PLOS ONE submissions requirements, in your Methods section, please provide additional information regarding the experiments involving animals and ensure you have included details on (1) methods of sacrifice, (2) methods of anesthesia and/or analgesia, and (3) efforts to alleviate suffering.***

[Response]

Additional information regarding the experiments was added to animals in 1.2.3.1 of the Methods section.

***3. We note that the grant information you provided in the ‘Funding Information’ and ‘Financial Disclosure’ sections do not match.***

***When you resubmit, please ensure that you provide the correct grant numbers for the awards you received for your study in the ‘Funding Information’ section.***

[Response]

This study was partly supported by non-restricted funds from Shabondama Soap Co., Ltd. and we did not receive any other grant or external funding. We declared it in the section of financial disclosure and competing interests in the editorial manager form.

***4. Thank you for stating in your Funding Statement:***

***“This study was partly supported by non-restricted funds from the Shabondama Soap CO., Ltd.”***

***Please provide an amended statement that declares *all* the funding or sources of support (whether external or internal to your organization) received during this study, as detailed online in our guide for authors at http://journals.plos.org/plosone/s/submit-now. Please also include the statement “There was no additional external funding received for this study.” in your updated Funding Statement.***

***Please include your amended Funding Statement within your cover letter. We will change the online submission form on your behalf.***

[Response]

Following your suggestion, we would like to modify the financial disclosure section as below.

Funding: This study was partly supported by non-restricted funds from the funder (Shabondama Soap Co., Ltd.) and we did not receive any other grant or external funding. The funding source provided support in the form of salaries for TKan, AM and TKawa. HM is the board of the funding source. However, he had no role in the study design, data collection and analysis, decision to publish, or preparation of the manuscript. The specific roles of these authors are articulated in the 'author contributions' section.

***5. Thank you for stating the following financial disclosure:***

***“This study was partly supported by non-restricted funds from the Shabondama Soap CO., Ltd. “***

***Please state what role the funders took in the study. If the funders had no role, please state: "The funders had no role in study design, data collection and analysis, decision to publish, or preparation of the manuscript."***

***If this statement is not correct you must amend it as needed.***

***Please include this amended Role of Funder statement in your cover letter; we will change the online submission form on your behalf.***

[Response]

As stated in the response to question 4, we corrected our financial disclosure section.

***6. Thank you for stating the following in your Competing Interests section: “no”***

***Please complete your Competing Interests on the online submission form to state any Competing Interests. If you have no competing interests, please state "The authors have declared that no competing interests exist.", as detailed online in our guide for authors at http://journals.plos.org/plosone/s/submit-now***

***This information should be included in your cover letter; we will change the online submission form on your behalf.***

[Response]

We modified the competing interests section as follows.

Competing interests: The funding source (Shabondama Soap Co., Ltd.) provided support in the form of salaries for TKan, AM and TKawa. This does not alter our adherence to PLOS ONE policies on sharing data and materials.

***7. Please provide a complete Data Availability Statement in the submission form, ensuring you include all necessary access information or a reason for why you are unable to make your data freely accessible. If your research concerns only data provided within your submission, please write "All data are in the manuscript and/or supporting information files" as your Data Availability Statement.***

[Response]

We modified the Data Availability Statement section as follows.

All relevant data are within the manuscript files.

***8. Please ensure that you refer to Figure 5 in your text as, if accepted, production will need this reference to link the reader to the figure.***

[Response]

We modified manuscript and mentioned Fig. 5 in section 2.5 “Questionnaires for clinical use,” line 3.

***9. Please include your tables as part of your main manuscript and remove the individual files. Please note that supplementary tables (should remain/ be uploaded) as separate ""supporting information"" files***

[Response]

We inserted the Tables 1-3 in the manuscript and removed them from the Figure file.

**Responses to Reviewer #1**

***Natural Soap is Less Toxic and More Biodegradable in Aquatic Organisms and Human Skin Cells than Synthetic Detergents and clinically efficient (suggest revise due to minor spelling and sentencing errors)***

[Response]

Thank you and the title is now corrected to “Natural Soap is Clinically Effective, Less Toxic, and More Biodegradable in Aquatic Organisms and Human Skin Cells than Synthetic Detergents”

***Materials & methods: Generally well written, suggest to provide abbreviation list. slight proofreading needed for the statistics section.***

[Response]

We provided the abbreviation list. We added explanations about statistics used for Toxicity effects on aquatic organisms and Human keratinocyte cytotoxic assay in the section of statistics.

***Results:***

**** Need a compiled list of abbreviations,***

[Response]

We provided the abbreviation list.

**** Once a term is abbreviated (i.e., sodium oleate (C18:1Na)), suggest to keep using the abbreviated version, instead of constantly keeping them in brackets.***

[Response]

We corrected our usage of abbreviated terms and brackets throughout our manuscript.

**** Suggest to review some sentences for grammatical errors. Images used are a bit blurry, suggest to substitute with at least 300dpi images.***

[Response]

We checked sentences of our manuscript. We reworked our figures to the Tiff files of at least 300 dpi.

**** Sections 2.2.1/2.2.2/2.2.3 : Result write-up should refer to the respective table immediately at the end of sentences or at the end of the paragraph, instead of at the end of the section.***

[Response]

We corrected the serial number of the Table which we referred to in these sections and inserted Table 2 after section 2.2.3. Table 2 shows the summary of the results of “2.2.1 Evaluation of algae,” “2.2.2 Evaluation of crustaceans” and “2.2.3 Evaluation of fish.” Each of these three sections explains the results throughout the section, so we think that the citation of Table 2 should be the current position.

**** Section 2.5: The results provided should refer to a graph/table instead of to a bibliographic reference [6]***

[Response]

We modified the manuscript and referred to Fig 5 in the explanation of results in section 2.5, line 3. The reference [6] was left to compare our results to the previous study.

***Discussion***

***- Generally well-written but had a heavy focus on results that have been presented well in the results section***

***- Suggest to make comparisons from the literature on similar studies and their findings.***

***- Suggest to add relevant environmental, economical, community & health impacts of the research***

***- Suggest to include the study strength and limitation as well as suggestions for future improvement etc***

[Response]

we are very much thankful for the reviewer #1’s careful and thoughtful comments and advice.

Comparisons with the literature have been included in both the *Introduction* and *Discussion*, and some limitations of this research are mentioned in the *Introduction*.

*Introduction*: Detergents claiming to contain natural and petrochemical-free ingredients have been found to contain petroleum-derived components, as determined by radiocarbon isotope (carbon-14) analysis. This finding may have implications for product safety and skin benefits [20].

Even though the addition of newly synthesized Evening Primrose amidopropyl sulfobetaine to surfactants containing SLS may potentially improve safety in SLS-based liquid washing soap, there is limited data on natural soap or its ingredients. [21].

*Discussion:*

A two-week exposure experiment was conducted to investigate the toxicity of linear alkylbenzene sulfonate (LAS) on five submerged macrophytes (four native and one exotic), focusing on their growth and physiological responses. The results showed that lower concentrations of LAS (<5 mg/L) slightly stimulated the growth of submerged macrophytes, while higher concentrations inhibited it. Increasing LAS levels led to a decrease in chlorophyll content, an increase in malondialdehyde content and peroxidase activity, and an initial rise in superoxide and catalase activities before a subsequent decline [33].

Gray water constitutes a significant fraction—50% to 80%—of total wastewater and has a cloudy appearance. It occupies an intermediate state between freshwater or drinking water and sewage. Recycling and reusing gray water are receiving increased attention due to its low concentrations of pathogenic microorganisms and nitrogen sources, despite its high phosphorus content.

One of the most problematic compounds in gray water is anionic surfactants, including linear alkylbenzene sulfonates (LAS) and branched alkylbenzene sulfonates (BAS), which are commonly used as ingredients in soaps and detergents.

A study investigating the molecular details underlying the biodegradation pattern of certain alkylbenzene sulfonates provided key insights. Two hydrogen bonds, essential for anchoring surfactants to the monooxygenase active site involved in the initial biodegradation step, were identified. These bonds determine how surfactants position themselves within the enzyme's hydrophobic pocket, thereby influencing the biodegradation rate in a structurally dependent manner.

These findings pave the way for the intelligent design of anionic surfactants that minimize their environmental impact in water and soil. To the best of our knowledge, this study represents a novel contribution toward the development of environmentally friendly surfactants with an enhanced likelihood of complete biodegradation and mineralization.[34]. Further studies using natural soap, which is similar to anionic surfactants in gray water, will help clarify its environmental effects.

Antiseptic synthetic hand soap often contains chloroxylenol (CHL), which serves as an alternative to triclosan and triclocarban—both of which have recently been banned in some countries. The more widespread use of CHL may significantly increase its presence and concentration in aquatic environments, potentially leading to ecological risks. Wastewater treatment plants (WWTPs) may act as point sources of CHL in natural environments due to its extensive discharge through urban waste streams.

Bioreactor operations and batch experiments were conducted to investigate the fate and effects of CHL and to elucidate the mechanisms underlying its degradation at various concentrations, ranging from environmentally relevant levels to high levels (0.5–5 mg L⁻¹). Bioreactors partially removed CHL (44–87%) primarily through biological processes. Microbial association networks, constructed using 16S rRNA gene sequencing data, revealed selective enrichment and a correlation between *Sphingobium* and CHL, suggesting its involvement in the biological breakdown of CHL through dehalogenation and ring hydroxylation pathways.

These findings provide valuable insights into the behavior and effects of CHL in activated sludge communities and offer important information for the sustainable management of CHL, which may become an emerging issue in the urban water cycle.[44].

***Reference:***

***Inadequate references for an original paper. Suggest to expand the discussion section which will eventually expand the numbers of references.***

***Conclusion:***

***- a good study and a generally well written manuscript, but needs improvement & expansion in the discussion to strengthen the manuscript further***

[Response]

we are very grateful for the reviewer #1’s kind suggestions and two references in *Introduction* and three references in *Discussion* are newly included in the revised format.

**Responses to Reviewer #2**

***1. Research Title: The title "Natural Soap is Less Toxic and More Biodegradable in Aquatic Organisms and Human Skin Cells than Synthetic Detergents and Clinically Efficient" suggests a conclusion that natural soap is indeed less toxic and more biodegradable, while the aim of the study is to explore these aspects. Would it be better to use a title such as: “Comparative Study on the Toxicity and Biodegradability of Natural Soap versus Synthetic Detergents in …” to better align with the study's objectives?***

[Response]

Thank you and the title is now corrected to “Natural Soap is Clinically Effective, Less Toxic, and More Biodegradable in Aquatic Organisms and Human Skin Cells than Synthetic Detergents”

***2. [Introduction]: This study has outlined various aspects of the differences between natural and synthetic soaps, including toxicity, biodegradability, effects on aquatic organisms, and impacts on human health and the environment. However, the research gap has not been explicitly stated. It would be beneficial to include a statement explaining what has not been thoroughly studied and why it is important.***

[Response]

we are very grateful for the reviewer #2’s careful comments and the lines and new references are added in Introduction.

Detergents claiming to contain natural and petrochemical-free ingredients have been found to contain petroleum-derived components, as determined by radiocarbon isotope (carbon-14) analysis. This finding may have implications for product safety and skin benefits [20].

Even though the addition of newly synthesized Evening Primrose amidopropyl sulfobetaine to surfactants containing SLS may potentially improve safety in SLS-based liquid washing soap, there is limited data on natural soap or its ingredients. [21].

***3. [Methodology]: In section 1.2.1, please explain the basis for determining the number of replicates, specifically the 3 replicates for treatments and 6 replicates for the control group.***

[Response]

We followed the guidelines of OECD TG 201. The guidelines state that the sample group should have "at least n=3 for each concentration" and the control group should have "minimum n=3, ideally twice the number of n for the sample group," so we set the sample to n=3 and the control to n=6. We inserted the explanation that we had followed OECD201 recommendations in section 1.2.1.

***4. [Methodology]: In section 1.4.3, concerning the LC50 equation [mM], is the use of log-log (double logarithmic) correct, or is it just a single logarithm? Please clarify.***

[Response]

The number of logs is one, so we corrected the number of logs in section 1.4.3. In addition, we also corrected the number of ln-ln in section 1.3.2 similarly.

***5. [Methodology]: In section 1.5, it is stated that the inclusion and exclusion criteria are in Table 3, but I could not find Table 3 in your submission. Please verify and include it.***

[Response]

We included Table 3 in section 1.5 and corrected the serial numbers of Table 3 to Table 1.

***6. [Methodology]: In section 1.5, it is mentioned that there are 50 study participants, including 4 other conditions. Please provide an explanation of these other conditions, as they represent variables in the study that need to be detailed.***

[Response]

We corrected the number of participants with other conditions from 4 to 14 and clarified other conditions and the explanation was written in section 1.5, line 3.

***7. [Methodology]: What type of statistics did you use in the analysis? Did you perform a test for differences in values? If so, please clarify which values were tested for differences.***

[Response]

We modified Statistic in **Materials and Methods** as follow:

All the data expressed mean ± standard deviation. For statistical analysis, the data of toxicity effects on aquatic organisms and biodegradability tests were analyzed in JMP 14 (SAS Institute, Inc., Cary, NC). In the Evaluation of algae, the linear regression analysis was used for EC_50_, and the one-way analysis of variance (ANOVA) was used for NOEC. In the evaluation of crustacean, probit analysis was used for EC_50_, and Dunn’s test was used for NOEC. In the evaluation of fish, binomial distribution was used. The data of LDH leakage and cell viability were analyzed by the one-way ANOVA. p-Values <0.05 were considered significant.

***8. [Results]: I also could not find Table 1 and Table 2 in your submission, making it impossible for me to check the contents of these tables. Kindly include the tables in the submission.***

[Response]

We included Table 1 and Table 2 in section 2.2 and section 2.3, respectively, and corrected the serial numbers of Table 1 and 2 to Table 2 and 3, respectively.

***9. [Results]: This study primarily focuses on assessing the toxicity of natural soap components on aquatic organisms, its biodegradability, and its cytotoxic effects on human keratinocytes. However, does this natural soap also provide superior antibacterial effectiveness compared to synthetic soap, given that the main function of soap is antibacterial, particularly in light of the COVID-19 pandemic background? It would be useful to include literature studies supporting the sustainability of your findings.***

[Response]

The comparative study on antibacterial effectiveness of natural soap and synthetic detergent has not been done. However, in the article shown as Reference 3, C18:1K, one of the components of natural soap, provided superior antibacterial effectiveness compared to alcohol-based disinfectant. In addition, C18:1K provided superior biofilm removal effectiveness to MRSA compared to synthetic detergents.

***10. [Conclusion]: I could not find a conclusion section in this submission. Please add a conclusion that includes the main findings and their implications.***

[Response]

We, authors, are very thankful for reviewer #2’s thoughtful indication and now we added the conclusion.

***11. Figure 5: The title is simply "Research of Questionnaires." The title of Figure 5 should be more descriptive, such as "Results of Questionnaire on the Effects of Natural Soap and Synthetic Detergents," and include explanations and units for each value from 1 to 4 to improve clarity.***

[Response]

We modified the title of Figure 5 and added the explanation of them in Figure 5. The Figure 5 is the result of the 4-point scale questionnaire, so these numbers have no units.

***12. I could not find an explanation of the abbreviation HBSS in the article. It would be helpful to define HBSS when it is first mentioned in the article, followed by the abbreviation in parentheses.***

[Response]

The abbreviation HBSS means Hanks' Balanced Salts Solution. We wrote the explanation in section 1.4, line 3. In addition, following to the suggestion of reviewer #1, we provided the abbreviation list including HBSS.
