# Supplementary material for: Natural soap is clinically effective and less toxic and more biodegradable in aquatic organisms and human skin cells than synthetic detergents
Source: PLoS One. 2025 Jun 18;20(6):e0324842. doi: 10.1371/journal.pone.0324842 (PMC12176228; doi:10.1371/journal.pone.0324842)
Supplement: Attachment — Submitted filename: Reviewer Comment, feb 17 2025.docx [file pone.0324842.s001.docx]

**Natural Soap is Less Toxic and More Biodegradable in Aquatic Organisms and Human Skin Cells than Synthetic Detergents and clinically efficient**

1. **Research Title**: The title "Natural Soap is Less Toxic and More Biodegradable in Aquatic Organisms and Human Skin Cells than Synthetic Detergents and Clinically Efficient" suggests a conclusion that natural soap is indeed less toxic and more biodegradable, while the aim of the study is to explore these aspects. Would it be better to use a title such as: “Comparative Study on the Toxicity and Biodegradability of Natural Soap versus Synthetic Detergents in …” to better align with the study's objectives?
2. **[Introduction]**: This study has outlined various aspects of the differences between natural and synthetic soaps, including toxicity, biodegradability, effects on aquatic organisms, and impacts on human health and the environment. However, the research gap has not been explicitly stated. It would be beneficial to include a statement explaining what has not been thoroughly studied and why it is important.
3. **[Methodology]**: In section 1.2.1, please explain the basis for determining the number of replicates, specifically the 3 replicates for treatments and 6 replicates for the control group.
4. **[Methodology]**: In section 1.4.3, concerning the LC50 equation [mM], is the use of log-log (double logarithmic) correct, or is it just a single logarithm? Please clarify.
5. **[Methodology]**: In section 1.5, it is stated that the inclusion and exclusion criteria are in Table 3, but I could not find Table 3 in your submission. Please verify and include it.
6. **[Methodology]**: In section 1.5, it is mentioned that there are 50 study participants, including 4 other conditions. Please provide an explanation of these other conditions, as they represent variables in the study that need to be detailed.
7. **[Methodology]**: What type of statistics did you use in the analysis? Did you perform a test for differences in values? If so, please clarify which values were tested for differences.
8. **[Results]**: I also could not find Table 1 and Table 2 in your submission, making it impossible for me to check the contents of these tables. Kindly include the tables in the submission.
9. **[Results]**: This study primarily focuses on assessing the toxicity of natural soap components on aquatic organisms, its biodegradability, and its cytotoxic effects on human keratinocytes. However, does this natural soap also provide superior antibacterial effectiveness compared to synthetic soap, given that the main function of soap is antibacterial, particularly in light of the COVID-19 pandemic background? It would be useful to include literature studies supporting the sustainability of your findings.
10. **[Conclusion]**: I could not find a conclusion section in this submission. Please add a conclusion that includes the main findings and their implications.
11. **Figure 5**: The title is simply "Research of Questionnaires." The title of Figure 5 should be more descriptive, such as "Results of Questionnaire on the Effects of Natural Soap and Synthetic Detergents," and include explanations and units for each value from 1 to 4 to improve clarity.
12. I could not find an explanation of the abbreviation HBSS in the article. It would be helpful to define HBSS when it is first mentioned in the article, followed by the abbreviation in parentheses.

**Thank you,**

**February 17, 2025.**
